# Open Ventral Hernia Repair: A Prospective Comparative Analysis of Onlay Versus Sublay Mesh Placement

**DOI:** 10.7759/cureus.113167

**Published:** 2026-07-22

**Authors:** Dravidsun M, Meenakshi Yeola, Srikanth M, Mallikarjun Gunjiganvi

**Affiliations:** 1 General Surgery, All India Institute of Medical Sciences, Mangalagiri, Mangalagiri, IND

**Keywords:** abdominal wall reconstruction, onlay mesh repair, seroma, sublay mesh repair, surgical site infection, ventral hernia

## Abstract

Background: Ventral hernia repair is among the most frequently performed general surgical procedures worldwide. Mesh-based repair has become the standard of care, but the optimal plane for mesh placement remains debated. This study compares outcomes of onlay and sublay techniques in open ventral hernia repair.

Methods: A prospective observational study enrolled 62 patients (31 per group) undergoing elective open ventral hernia repair at a tertiary care institute in India over 18 months. Postoperative wound complications (seroma, surgical site infection (SSI)), operative duration, hospital stay, and pain scores (Visual Analog Scale, postoperative days (POD) 1, 3, 5) were compared. Statistical analysis used the Mann-Whitney U test, the Chi-square/Fisher exact tests, and binary logistic regression (SPSS Statistics version 30 (IBM Corp., Armonk, NY, USA)).

Results: Groups were comparable in age (onlay 51.3±14.2 vs sublay 51.7±11.0 years; p = 0.897), gender, body-mass index (BMI), and comorbidities. Seroma occurred significantly more often in the onlay group (32.3% vs 3.2%; p = 0.003). SSI rates were higher in onlay patients (25.8% vs 16.1%) but did not reach statistical significance (p = 0.349). Operative time was significantly shorter in the onlay group (94.4 vs 126.7 minutes; p<0.001). Onlay patients had less pain on POD 1 (p = 0.042). Median hospital stay was similar (9 vs 8 days; p = 0.155). No hematoma, mesh infection, or wound dehiscence occurred in either group. Subcutaneous fat thickness was an independent predictor of seroma (p = 0.026).

Conclusion: Both techniques are safe for open ventral hernia repair. Sublay mesh placement significantly reduces seroma formation and trends toward fewer SSIs, while onlay repair offers a shorter operative time and less immediate postoperative pain. Surgical decision-making should be individualized based on anatomical factors, comorbidities, and intraoperative findings.

## Introduction

Ventral hernias encompass a spectrum of defects in the anterior abdominal wall, including umbilical, epigastric, and incisional hernias. Incisional hernias alone occur in up to 11-20% of patients following laparotomy [1], with an estimated 350,000 to 500,000 ventral hernia repairs performed annually worldwide [2]. These conditions impose considerable morbidity, impair quality of life, and represent a significant healthcare burden. The introduction of prosthetic mesh fundamentally transformed hernia surgery, reducing recurrence rates compared with primary suture closure [3]. Among open mesh techniques, the onlay (suprafascial) and sublay (retrorectus) approaches are most widely employed. In the onlay technique, mesh is positioned above the anterior rectus sheath following primary fascial closure, requiring minimal dissection but placing the prosthesis in proximity to poorly vascularized subcutaneous tissue, a setting associated with higher rates of seroma and infection [4,5]. The sublay (Rives-Stoppa) technique places mesh in the well-vascularized retrorectus plane between the posterior sheath and the rectus abdominis muscle, affording greater mechanical support and lower complication rates at the cost of increased operative complexity and duration [1,4]. Despite accumulating evidence favoring sublay repair, most comparative studies are retrospective, single-center, or limited by small cohort sizes and variable outcome definitions. Data from resource-constrained settings, where patient demographics, nutritional status, and practice patterns differ markedly from those of Western cohorts, remain scarce. The primary objective of this prospective observational study was to compare postoperative wound complications between onlay and sublay open ventral hernia repair; secondary objectives included comparison of operative duration, postoperative pain, and length of hospital stay in a tertiary care teaching hospital in India.

## Materials and methods

Study design and setting

A hospital-based, prospective, observational study was conducted over 18 months in the Department of General Surgery at the All India Institute of Medical Sciences (AIIMS), Mangalagiri, Andhra Pradesh, India. The study was approved by the Institutional Ethics Committee (AIIMS/MG/IEC/2023-24/53), and written informed consent was obtained from all participants.

Patients more than 18 years old who were admitted for elective open ventral hernia repair were enrolled for this research consecutively. Patients requiring emergency intervention for obstruction, incarceration, or strangulation, and those with gross ascites, were excluded.

**Figure 1:**
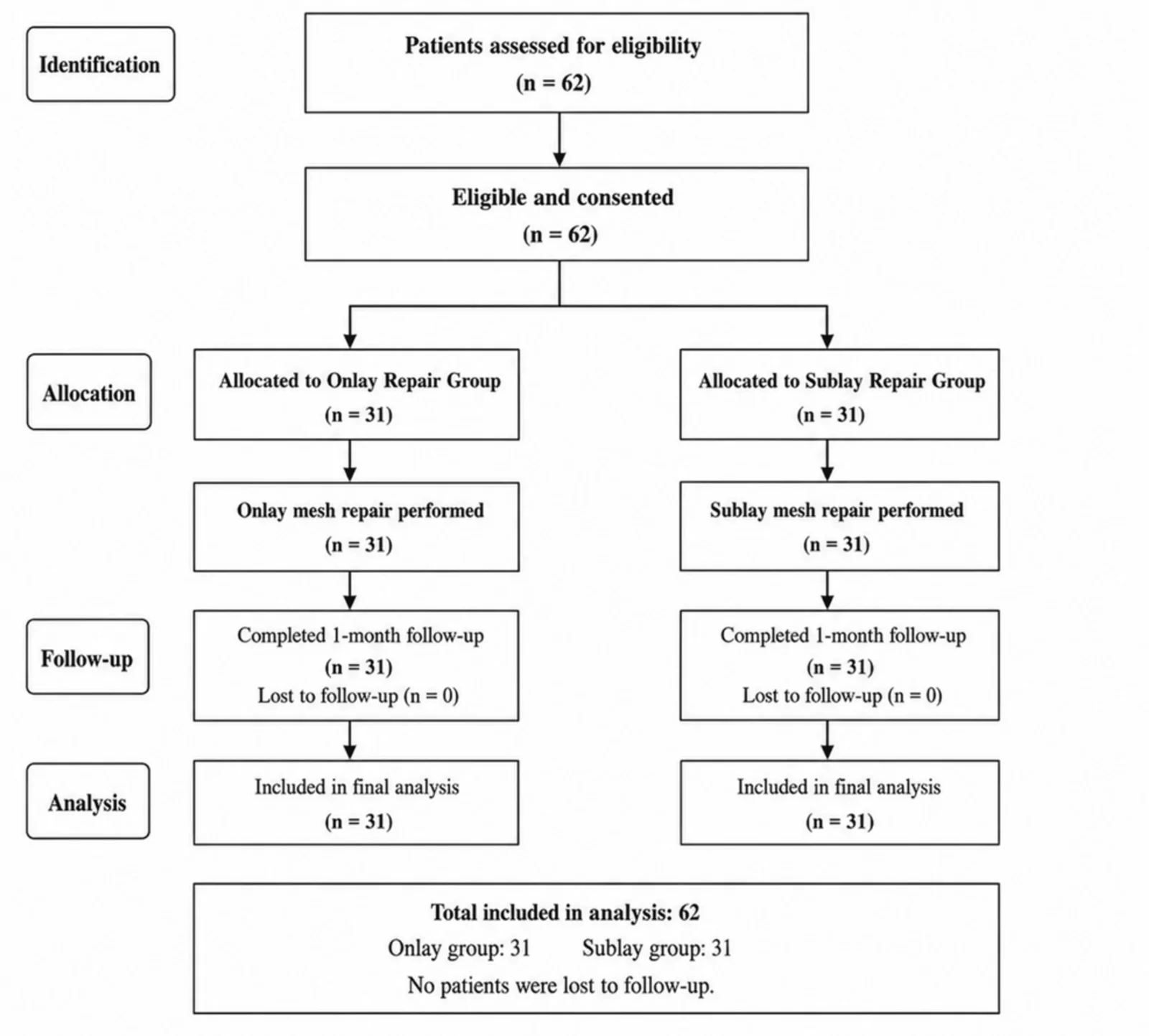
STrengthening the Reporting of OBservational studies in Epidemiology (STROBE) flow diagram [7]

Sample size was determined using OpenEpi v3.01 [6], based on an anticipated postoperative seroma rate of 39.4% (onlay) versus 9.0% (sublay) from prior literature, with 5% alpha error and 80% power, yielding a minimum of 31 patients per group (Figure 1).

Intraoperative allocation to onlay or sublay repair was determined by the operating surgeon based on anatomical findings, clinical considerations, and individual surgeon preference, rather than an experimental randomization protocol. In sublay repair, the retrorectus plane was developed bilaterally; the peritoneum with the posterior rectus sheath was closed with continuous 2-0 Vicryl (Ethicon, Raritan, NJ, USA). A polypropylene mesh (3-5 times the defect area) was fixed in the retrorectus plane with 2-0 Prolene (Ethicon). Two 14F closed suction drains (retrorectus and anterectus) were placed. The anterior rectus sheath was closed with continuous 1-0 Prolene. In onlay repair, the defect was closed with 1.0 Prolene; the anterectus plane was created, and the mesh was fixed with 2-0 Prolene. A single 14F drain was placed in the anterectus plane. Skin closure was performed with 2-0 Ethilon (Ethicon) and skin staplers in both groups. Antibiotic prophylaxis consisted of three doses of cefuroxime 1.5 g intravenous (one preoperatively, two postoperatively), followed by oral cefuroxime 500 mg twice daily for three days, as per the institutional antibiotic policy. Postoperative analgesia comprised paracetamol 1 g intravenous every eight hours and tramadol 50 mg intravenous on demand for the first two days, transitioning to oral aceclofenac + paracetamol.

Outcome assessment

The primary outcomes were seroma and surgical site infection (SSI) rates. Secondary outcomes included hematoma, wound dehiscence, mesh infection, operative duration, postoperative hospital stay, and pain scores. Seroma was defined as a clinically palpable subcutaneous collection following drain removal, confirmed by ultrasonography when required. SSI was classified according to CDC/National Healthcare Safety Network (NHSN) criteria [2]. Pain was assessed using the visual analog scale (VAS 0-10) on postoperative days (POD) 1, 3, and 5. A follow-up was conducted weekly for one month.

Statistical analysis

Data were analyzed using SPSS Statistics version 30 (IBM Corp., Armonk, NY, USA). Categorical variables were compared with Chi-square or Fisher's exact tests; continuous variables with an independent T-test or Mann-Whitney U test based on normality. Multivariable binary logistic regression identified independent predictors of seroma and SSI. Results are expressed as mean ± SD, median (IQR), frequency (%), or odds ratio (95% CI). A p-value <0.05 was considered statistically significant.

## Results

Baseline characteristics

A total of 62 patients (31/group) were enrolled. The groups were well-matched for age (onlay 51.31 ± 14.20 vs sublay 51.74 ± 10.96 years; Student's t= -0.13, df = 60, p = 0.897, 95% CI of difference: -7.02 to 6.16), female gender (74.2% vs 80.6%; Pearson Chi-square = 0.37, df = 1, p = 0.544), BMI (30.55 ± 4.18 vs 29.30 ± 3.79 kg/m^2^; t = 1.23, df = 60, p = 0.224, 95% CI of difference: -0.78 to 3.28), and comorbidities including obesity (54.8% vs 41.9%; Chi-square = 1.03, df = 1, p = 0.309), diabetes mellitus (19.4% vs 22.6%; Chi-square = 0.10, df = 1, p = 0.755), and hypertension (41.9% vs 25.8%; Chi-square = 1.80, df = 1, p = 0.180). A history of prior SSI was more common in the onlay group (12.9% vs 0%; Fisher's exact test, p = 0.039). Incisional hernia was the most prevalent type in both groups (58.1% onlay, 71.0% sublay). European Hernia Society (EHS) classification differed significantly between groups (Chi-square = 8.31, df = 3, p = 0.040), with M4W2 being most common in both (38.7% vs 35.5%); M4W1 was disproportionately represented in the sublay group (35.5% vs 3.2%) [8]. Subcutaneous fat thickness was significantly greater in the onlay group (median 3 cm (IQR 2-3) vs 2 cm (IQR 1.5-2.5); Mann-Whitney U = 232.0, Standardized Z = -3.28, p = 0.001). Defect width did not differ significantly (4.67 +/- 2.77 vs 3.69 +/- 1.85 cm; t = 1.63, df = 60, p = 0.107, 95% CI of difference: -0.22 to 2.18). The primary intraoperative determinant of technique was surgeon decision (54.8% in both groups), while difficult plane dissection drove onlay selection in 45.2% of onlay cases, and successful retrorectus plane development prompted sublay repair in 38.7% of sublay cases (Chi-square = 31.42, df = 3, p<0.001) (Table 1).

**Table 1:** Baseline characteristics of study participants * Statistically significant (p <0.05). SC- subcutaneous; SSI- surgical site infection; IQR- interquartile range

Variable	Onlay (N/%)/ Mean ± SD	Sublay (N/%)/ Mean ± SD	P value
Total participants	31 (100%)	31 (100%)	-
Age (years)	51.31 ± 14.20	51.74 ± 10.96	0.897
Female gender	23 (74.2%)	25 (80.6%)	0.544
BMI (kg/m²)	30.55 ± 4.18	29.30 ± 3.79	0.224
Obesity	17 (54.8%)	13 (41.9%)	0.309
Diabetes mellitus	6 (19.4%)	7 (22.6%)	0.755
Hypertension	13 (41.9%)	8 (25.8%)	0.180
Prior SSI	4 (12.9%)	0 (0.0%)	0.039*
SC fat thickness (cm), median (IQR)	3 (2-3)	2 (1.5-2.5)	0.001*
Defect width (cm)	4.67 ± 2.77	3.69 ± 1.85	0.107

Postoperative wound complications

Seroma formation was significantly more common in the onlay group: 10/31 patients (32.3%) versus 1/31 patients (3.2%) in the sublay group (p = 0.003). No hematoma, mesh infection, or wound dehiscence occurred in either group. Superficial SSI developed in 8/31 onlay patients (25.8%) and 5/31 sublay patients (16.1%); this difference was not statistically significant (p = 0.349).

In multivariable binary logistic regression, greater subcutaneous fat thickness was the only statistically significant independent predictor of seroma (aOR = 2.41, 95% CI: 1.11 to 5.23, Wald = 4.96, p = 0.026). Diabetes mellitus (aOR = 0.38, 95% CI: 0.13 to 1.10, Wald = 3.21, p = 0.073) and longer operative duration (aOR = 1.03, 95% CI: 0.99 to 1.07, Wald = 2.95, p = 0.086) showed non-significant trends. For SSI, no variable reached significance; longer operative time (aOR = 1.02, 95% CI: 0.99 to 1.05, Wald = 3.11, p = 0.078) and older age (aOR = 1.04, 95% CI: 0.99 to 1.09, Wald = 2.91, p = 0.088) showed marginal trends. The sublay technique was associated with a non-significant trend toward lower SSI risk (aOR = 0.44, 95% CI: 0.15 to 1.25, Wald = 2.33, p = 0.127) (Table 2).

**Table 2:** Postoperative outcomes * Statistically significant (p<0.05). SSI- surgical site infection; VAS- visual analog scale; IQR- interquartile range; POD- postoperative day

Outcome	Onlay (N/%)/ Mean ± SD	Sublay (N/%)/ Mean ± SD	P value
Seroma	10 (32.3%)	1 (3.2%)	0.003*
SSI	8 (25.8%)	5 (16.1%)	0.349
Hematoma	0	0	-
Wound dehiscence	0	0	-
Mesh infection	0	0	-
Operative time (minutes)	94.4 ± 31.3	126.7 ± 24.5	<0.001*
Hospital stay (days), median (IQR)	9 (7-13)	8 (6-10)	0.155
VAS pain score POD 1, median (IQR)	4 (4-4)	4 (4-6)	0.042*
VAS pain score POD 3, median (IQR)	2 (2-4)	4 (2-4)	0.092
VAS pain score POD 5, median (IQR)	2 (1-3)	2 (1-3)	0.173

Operative duration and hospital stay

Mean operative duration was significantly shorter in the onlay group (94.42 ± 31.3 vs 126.71 ± 24.5 minutes; Student's t = -4.38, df = 60, p < 0.001, 95% CI of difference: -47.05 to -17.53 minutes). Median postoperative hospital stay was nine days (IQR 7-13) in the onlay group and eight days (IQR 6-10) in the sublay group, with no statistically significant difference (Mann-Whitney U = 398.5, Standardized Z = -1.42, p = 0.155).

Postoperative pain

On POD 1, the onlay group had lower pain variability (median 4, IQR 4-4 vs 4, IQR 4-6; Mann-Whitney U = 345.5, Standardized Z = -2.03, p = 0.042). By POD 3, pain scores converged (onlay median 2 vs sublay 4; U = 372.0, Z = -1.68, p = 0.092) and were identical by POD 5 (median 2 in both; U = 465.0, Z = -0.21, p = 0.173).

## Discussion

This prospective study comparing onlay and sublay open ventral hernia repair in an Indian tertiary care setting demonstrates that sublay mesh placement is associated with a significantly lower incidence of postoperative seroma, corroborating prior meta-analyses [1,5]. The clinically important seroma reduction (32.3% to 3.2%; p = 0.003) is consistent with the mechanistic explanation that the retrorectus position eliminates the large dead space inherent to the suprafascial plane, reduces shear forces on the mesh, and places the prosthesis within a well-vascularized tissue environment less prone to fluid accumulation [4,5]. Greater subcutaneous fat thickness was identified as the sole independent predictor of seroma on multivariable analysis (p = 0.026), consistent with evidence linking obesity and thick abdominal panniculus to increased lymphatic disruption and dead space formation [9,10]. Notably, subcutaneous fat was significantly greater in the onlay group (median 3 vs 2 cm; p = 0.001), likely reflecting the intraoperative tendency to select onlay repair when the retrorectus plane is difficult to dissect, as confirmed by our procedural data. This selection bias represents a key limitation of observational hernia research and is inherent to the present design.

The SSI rate was numerically higher in the onlay group (25.8% vs 16.1%) but did not reach statistical significance (p = 0.349). This aligns with the systematic review by Pereira et al., and the Brazilian cohort study by Martins et al., both of which found no statistically significant inter-technique difference in SSI [10,11]. The randomized controlled trial by Kancharla et al., however, reported significantly higher SSI in the onlay group (p<0.02) [12], attributing this to more extensive skin flap elevation required for suprafascial mesh placement, a mechanistic pathway that may be more pronounced with larger defects or more obese patients than those in our cohort. This study was adequately powered to detect the anticipated seroma difference but was not powered to detect modest differences in SSI rates; a larger sample would be required to resolve this question definitively.

The significantly longer operative time in the sublay group (126.7 vs 94.4 minutes; p<0.001) reflects the technical demands of bilateral retrorectus dissection, posterior sheath closure, and management of incidental peritoneal tears, particularly in patients with previous abdominal surgery (80.6% of sublay cases vs 67.7% onlay). Sevinc et al. reported a similar directional difference (73.9 versus 56.7 minutes) [13], as did several other centers, confirming this as a reproducible finding rather than an institutional artifact.

POD 1 pain scores were significantly lower in the onlay group (p = 0.042), plausibly reflecting the more extensive muscular dissection of retrorectus repair. This advantage was transient, with convergence by POD 3 and equivalence by POD 5. Kancharla et al., similarly, observed less early pain in the onlay group [12], while Den Hartog et al. reported less pain with sublay repair, a discrepancy that may relate to differences in analgesia protocols, patient populations, and drain management [14]. Hospital stay did not differ significantly between the groups in this research study (9 versus 8 days; p = 0.155), in contrast to an Indian randomized controlled trial, which found a significantly shorter stay with sublay repair, attributing this to higher SSI-related prolongation in the onlay group [15,16]. The absence of this effect in this cohort may reflect our lower overall SSI rates or differences in drain management protocols.

Limitations

This research is an observational study with an intraoperative technique selection that introduces selection bias. Patients allocated to onlay repair had thicker subcutaneous fat, a higher prevalence of prior SSI, and more complex hernia morphology according to the EHS classification, which might independently influence outcomes. Second, the single-center design may limit generalizability. The study's limited sample size and short follow-up duration lacked the power to detect modest differences in low-frequency outcomes, such as mesh infection or long-term recurrence. These limitations notwithstanding, the prospective design, standardized surgical protocols, uniform outcome definitions per CDC criteria, and complete follow-up represent methodological strengths relative to many prior studies in this field.

## Conclusions

Both onlay and sublay mesh techniques represent safe and effective approaches to open ventral hernia repair. Sublay placement significantly reduces seroma formation and may trend toward fewer SSIs, while the onlay technique offers a shorter operative time and less immediate postoperative pain. The choice of technique should be individualized, with sublay repair preferred where anatomical conditions permit, given its superior complication profile. Prospective multicentre trials with longer follow-up and quality-of-life endpoints are required to establish definitive evidence-based recommendations, including comparisons with minimally invasive approaches.
